# Specific Remedies in Infectious Diseases; Translated from the German

**Published:** 1894-02

**Authors:** 


For Texas Medical Journal.

### SPECIFIC REMEDIES IN INFECTIOUS DISEASES.

(Translated from Berliner Klinische Wochenschrift, Nov. 27, 1893.)

ALMOST from the very moment that the bacteriological methods of investigation proved the participation of specific micro-organisms in the production of infectious diseases, dates the attempt to meet these producers of disease by specific remedies. The hope to destroy the bacillus of typhoid fever or the pneumococcus by "internal asepsis," has not been materialized up to the present. Much more promising appeared the attempt to introduce into the system the products of the microbe, imitating the natural processes of recovery, so as to kill the parasites by their own poisons. Experiments on animals gave many reasons to expect good from such a rational method. It even appeared possible that the highest aim of therapeutics, a reliable prophylaxis, could be finally obtained, as a variety of facts brought out the feasibility of immunization against certain bacterial poisons. These experiments have been conducted up to date in two different directions. The simplest way seemed to be to use the poisonous bacterial substance itself as the remedy, injecting certain quantities of living or dead cultures into the body, with the view, either to heal the sick by destroying the immigrated bacteria, or to immunize the system of the healthy against them before their invasion. More complicated, but more in conformity with the natural processes was the method to use the blood-serum of immunized animals for both purposes, a method which is foremost represented by Behring's known experiments with the serum of diphtheritic animals. It is for our present purpose, immaterial whether the success of this method is explained by the influence of specific auto-poisons, or whether it is ascribed to an increased energy of the tissues, caused by specific irritating substances.

The intention of these remarks is to say a few words about the influence of the bacteria themselves, and their derivatives, excluding consideration of the healing serum.

Practical attention to this form of anti-parasitical treatment was inaugurated by Koch's discovery, that certain derivates of the bacillus tuberculosis, when introduced into the diseased system, will produce a general fever, as well as a local effect—namely, increased inflammation. This observation was broadened, and the apparent specific effect was made so much more plausible, as it was found that a body could be extracted from the tuberculin that would produce the local effect without any general pyrogenic symptoms. However, further researches in this very direction, weakened the assumption of such specific influences considerably. Roemer and Buchner were the first to show that those very effects were common to other bacterial proteins of entirely different origin. It was even found that the effect attributed to the tuberculin was not at all a specific feature of proteins—that altogether different chemical substances are just as well able to produce similar, more or less intensive processes. Liebreich proved it for cantharidin, Von Mosetig for tenkrin, Von Hebra for sulfallyl-urea, and a recently published paper by Spiegler, reports a large number of such substances like thiophen, benzol, aceton, tanrin, propylamin and others, of which the ones containing amin, have an exceptionally strong influence. Concededly, the practical therapeutic side of the question has lost a good deal of interest.

Of greater importance seem to be the results recently obtained in the studies of typhoid fever. E. Fraenkel, of Hamburg, was the first to use hypodermic injections of sterilized thymus-bouillon cultures of the typhoid bacillus on man, prompted by the experiments of Brieger, Kitasato and Wassermann on animals. The effect was surprisingly good. On the third day of treatment the temperature fell; from the start the continuous fever became remittent, and in a very short time full apyrexia and convalescence set in. This excellent result was apparently a splendid proof of the value of specific treatment, and, no doubt, it would have been used as a strong argument in favor of this method, had not a contemporary report by Rumpf changed the situation altogether. Rumpf obtained the very same favorable results, not from injections of the typhoid fever bacillus culture, but from those of the pyocyanic bacillus; whilst, it is true, streptococcus bouillon was a failure. He, too, was unmistakably successful, and insists that no other treatment ever proved so efficacious. In fact, a cutting short of the disease to from six to eight days hardly admits a doubt of the injections being the real cause of the brilliant cures. Indeed, it speaks against the specific effect of Fraenkel's treatment. Buchner concludes from his experiments that there is no autotoxic influence to be surmised, nor immunization; that rather by certain chemical substances an increased reaction of the diseased tissues is caused (perhaps by chemotaxis of the leucocytes), which process he calls an energetic mobilization of all available forces of the organism. It may be added that, according to Buchner's statement, the thymus extract by itself acts chemo-tactically on leucocytes, and thus causes fever.

Still more of a basis in the study of the effects of different bacterial products have we lately gained by the researches in cholera. As known, the differentiation of the cholera bacillus is partially based upon the results of experimental infection of animals. Intraperitoneal injections of certain quantities of a culture will kill guinea pigs, with more or less of the picture of Asiatic cholera. The discovery of Klein, corroborated by Sobernheim, that altogether different species of bacteria (the proteus vulgaris, the prodigiosus, the coli communis, the Finkler's, the hay fever and typhoid fever bacillus) produce exactly the same symptom—complex, caused not a little consternation. Sobernheim states that not only the symptoms, but even the post-mortem findings were exactly the same as after injection of the cholera bacillus culture.

But still more important are the results of the researches of the last named experimenters in regard to the much ventilated question of immunizing inoculations against cholera. It has been found that animals which had been inoculated with the different named bacilli, or their heated cultures, became just as immune against cholera infection as if they had been inoculated with the cholera bacillus itself. From such facts, it must be concluded that neither the morbid symptoms in animals after intraperitoneal injections of the cholera bacillus, nor the immunity following them, are of any specific nature, and that rather the effects of the products of certain very different bacteria are identical.

